# Challenges and Opportunities of Bacterial Vaccines as Alternatives to Antimicrobials in Swine Health Management: Insights from U.S. Veterinarians

**DOI:** 10.3390/pathogens14111113

**Published:** 2025-11-01

**Authors:** Xirui Zhang, Danqin Li, Michael D. Apley, Locke Karriker, Joseph F. Connor, Corinne Bromfield, Jordan T. Gebhardt, Brian Lubbers, Hatem Kittana, Dustin Pendell, Rachel Madera, Nina Muro, Aidan Craig, Brooke Shenkenberg, Yuzhen Li, Lihua Wang, Jishu Shi

**Affiliations:** 1 Center on Biologics Development and Evaluation, College of Veterinary Medicine, Kansas State University, Manhattan, KS 66506, USA; xzr92@missouri.edu (X.Z.); danqinli@vet.k-state.edu (D.L.); rachelmadera@vet.k-state.edu (R.M.); nina14@vet.k-state.edu (N.M.); aidan3@vet.k-state.edu (A.C.); shenky@vet.k-state.edu (B.S.); yuzhen@vet.k-state.edu (Y.L.); 2 Department of Anatomy and Physiology, College of Veterinary Medicine, Kansas State University, Manhattan, KS 66506, USA; mapley@vet.k-state.edu; 3 College of Veterinary Medicine, University of Missouri, Columbia, MO 65211, USA; bromfieldc@missouri.edu; 4 Department of Veterinary Diagnostic and Production Animal Medicine, College of Veterinary Medicine, Iowa State University, Ames, IA 50011, USA; karriker@iastate.edu; 5 Carthage Veterinary Service, LTD., Carthage, IL 62321, USA; connor@hogvet.com; 6 Department of Diagnostic Medicine/Pathobiology, College of Veterinary Medicine, Kansas State University, Manhattan, KS 66506, USA; jgebhardt@vet.k-state.edu (J.T.G.); hkittana@vet.k-state.edu (H.K.); 7 Department of Clinical Sciences, College of Veterinary Medicine, Kansas State University, Manhattan, KS 66506, USA; blubbers@vet.k-state.edu; 8 Department of Agricultural Economics, College of Agriculture, Kansas State University, Manhattan, KS 66506, USA; dpendell@k-state.edu

**Keywords:** antimicrobial resistance, alternatives, bacterial vaccines, swine health, veterinarians, challenges, opportunities

## Abstract

Antimicrobial resistance (AMR) is a significant global health concern, and the use of antibiotics in livestock, including swine production, is a major contributor. Vaccines offer a promising alternative for controlling bacterial infections in pigs, but their widespread use is often hindered by biological, economic, and practical challenges. This study surveyed U.S. swine veterinarians to identify which bacterial diseases require better vaccines and to understand the barriers to their adoption. Nineteen veterinarians with an average of 24.7 years of experience were surveyed across 21 states. The results identified *Streptococcus suis*, *Escherichia coli*, *Mycoplasma hyopneumoniae*, and *Glaesserella parasuis* as the most critical pathogens needing improved vaccines. Veterinarians anticipated significant improvements in vaccine efficacy for *S. suis* and *E. coli* during the nursery stage and expressed a willingness to pay 1.8 and 1.9 times their current prices, respectively. While expectations for *M. hyo* vaccine improvements were not significant, veterinarians expressed the highest willingness to pay (4.2 times the current price), citing the potential for disease eradication. This research highlights that developing effective vaccines for *S. suis* and *E. coli* should be the most urgent priority due to their significant economic impact and rising AMR concerns. However, *M. hyo* vaccine development holds the most economic potential due to the possibility of eradication. Our research provides a roadmap for future efforts to combat AMR in the swine industry, emphasizing key economic, policy, and educational considerations for successful vaccine implementation.

## 1. Introduction

Antimicrobials, hailed as one of the 20th century’s most significant medical breakthroughs, encompass a wide range of substances capable of destroying or inhibiting microbial growth [1]. Their widespread application has saved countless human and animal lives from infectious diseases, profoundly impacting public health, the global economy, agriculture, and animal welfare [1,2]. However, the efficacy of antimicrobials is increasingly threatened by antimicrobial resistance (AMR). This phenomenon occurs when microorganisms evolve under the selective pressure of antimicrobial use, rendering these life-saving drugs ineffective. Decades of widespread misuse and overuse of antimicrobials have largely fueled this resistance [3]. Antibiotics, a major category of antimicrobials targeting bacteria, are the most significant contributors to the AMR crisis. The development of new and effective antibiotics has been slow, failing to keep pace with the rapid rise in AMR. According to the WHO, between 2017 and 2022, only 12 new antibiotics were approved, 10 of which had documented resistance mechanisms [4]. The UK Government-commissioned report further estimates that by 2050, AMR could threaten 10 million lives globally, cause $100 trillion of economic losses, and push countless more into poverty [3,5]. Consequently, safeguarding antimicrobials as a limited resource has become a recognized priority for governments and international organizations.

Statistically, the majority of antimicrobials are being used in livestock production. In developed countries, an estimated 50–80% of antibiotics are consumed by livestock [6]. This trend is particularly pronounced in the swine industry, where primary and secondary bacterial infections are common and frequently lead to substantial economic losses. Antibiotics, as effective countermeasures that directly act against bacteria, have naturally become one of the most relied-upon tools to combat or prevent these bacterial diseases in swine practice [7,8,9]. In the U.S., despite the established strong awareness within the swine veterinary community and the ongoing emphasis on antimicrobial stewardship to reduce reliance on antibiotics and alleviate the development of AMR, the amount of antibiotics used each year remains alarmingly high. A 2022 report by the U.S. FDA revealed that 43% of all medically important antimicrobial drugs sold are for use in swine production, ranking the highest among all food-producing animals [10]. This highlights the critical need to continually explore and evaluate both novel and traditional strategies to further reduce antimicrobial use in swine production.

Experts have proposed numerous alternatives and strategies to reduce antimicrobial use, addressing various aspects of animal health management. Among these, vaccines are consistently regarded as one of the most effective and practical options, alongside biosecurity, improved management practices, nutrition, water quality, and environmental factors [3,11,12]. Aligning with the WHO’s global action goal on AMR, the OIE has also stated that veterinary vaccines represent the most cost-effective medical intervention available to address the threat of AMR [13,14,15]. In swine health management, research shows that vaccines rank among the top five methods in both effectiveness and feasibility as alternatives to antimicrobials [11]. Several studies have demonstrated that using vaccines has significantly reduced antimicrobial consumption while improving outcomes such as lower mortality rates and better daily weight gain [16,17]. However, while vaccines for certain diseases in the swine industry have shown significant success, many others—especially those targeting bacteria—still fall short of meeting the standard required to serve as viable alternatives to antibiotics, thus limiting further success in the reduction in antibiotic use [18]. This often caused by limited understanding of bacterial physiology and pathobiology, leading to suboptimal vaccine efficacy [19]. Additionally, various factors affecting animal production, such as stress, fecal microbiota, genetics, and maternal-derived antibodies (MDAs), also significantly influence vaccine outcomes [20,21,22]. Beyond the direct interactions of vaccine and animal, real-world production considerations like cost-effectiveness, vaccine pricing, time and labor expenses, vaccine availability, potential side effects, and development cost all influence stakeholders’ operational decisions and ultimately impact the balance between antimicrobial use and vaccine utilization.

This study aims to investigate the perspectives of practicing swine veterinarians in the U.S. to identify urgently needed vaccines, pinpoint shortcomings of existing ones, and understand expectations for future vaccine developments. By analyzing the current state of disease prevention and control for different bacterial pathogens, identifying factors driving vaccine urgency, and proposing solutions aligned with production needs, this research seeks to provide a multifaceted discussion on the feasibility of bacterial vaccines replacing antibiotics in swine production. Furthermore, it explores the economic, policy, and educational factors crucial for successful vaccine implementation, offering potential research directions and supporting decision-making processes to effectively mitigate antimicrobial resistance in the swine industry.

## 2. Materials and Methods

### 2.1. Survey Design

Our survey was implemented using the Qualtrics software, Copyright© 2024 Qualtrics, Provo, UT, USA. Available at http://www.qualtrics.com (accessed on 10 August 2024), access with license through the Kansas State University (KSU), KS, USA. The study, approved by the Kansas State University Research Compliance Office (Protocol # IRB-12219), followed all relevant laws and guidelines in its procedures. We maintained the privacy rights of all human subjects involved in the experiments and we ensured that informed consent was obtained from each participant prior to their involvement.

The survey’s design commenced in March 2024, guided by three veterinary food-animal medicine specialists from Kansas State University’s College of Veterinary Medicine, to maintain the integrity of evidence-based methodology. A pretest and revisions were conducted in May to refine the questions before finalizing the survey. The finalized version of the survey was launched in June 2024, distributed to a total of 202 contacts, and closed for response collection in August.

The survey was designed to be conducted online, enabling respondents to participate via either an internet-connected PC or smartphone. All survey responses were anonymous, no personal information was collected, and no screening of respondents based on demographic data (sex, age, or ethnicity) was performed. As an incentive for participation, respondents could voluntarily choose to receive a $30 gift card at their discretion.

### 2.2. Survey Dissemination

The survey was disseminated through two channels: the Qualtrics distribution system or direct emails sent by researchers. A periodic reminder was sent one week after the initial distribution. Respondents’ contact information was obtained through several channels, with most contacts sourced from the AASV website, while others were provided by authors or found online via cooperations’ websites. Apart from verifying that they were practicing veterinarians, no additional criteria were used to filter respondents, ensuring that the data represented the broadest range of perspectives.

The collection of our survey data commenced on 18th June 2024, and concluded on 9th August 2024. All respondents were licensed DVM practitioners in North America. Some contacts who were accidentally contacted were, in fact, swine nutritionists or economists, and they were ultimately asked not to participate in the survey to ensure the source of our information comes from only practicing veterinarians. To ensure no instances of duplicated responses, we have double-checked and confirmed the IP address of each response we received.

### 2.3. Question Development

**Figure 1:**
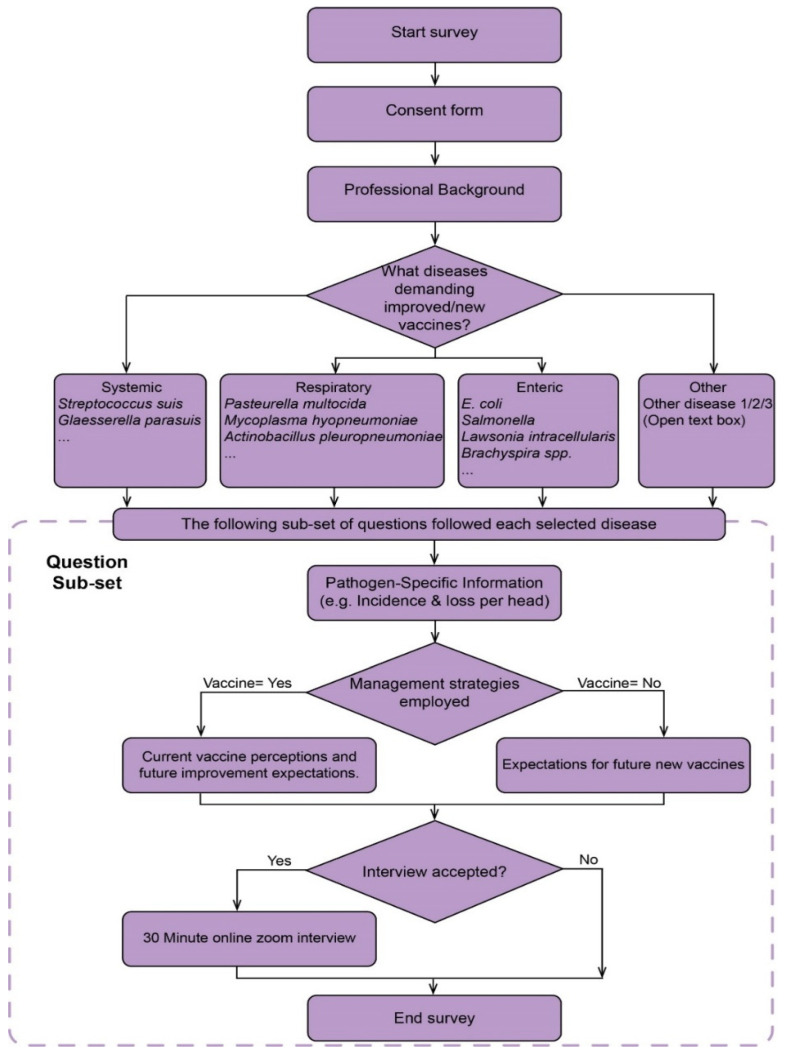
Diagrammatic representation of the survey process.

The survey comprises 5 parts. The workflow of our survey is illustrated in Figure 1.

(i) Participants’ professional background. The survey begins by gathering basic professional information from respondents, mainly their years of experience in the swine industry, the number of pigs overseen, and their geographic location. No personal information such as name, age, or address was collected.
(ii) Identifying diseases demanding improved/new vaccines. The next section focuses on identifying the most pressing bacterial diseases in swine production. The following subsets of questions of the survey will be based on the pathogens selected here. The selection and classification of the listed key candidate diseases are primarily based on the Report of the Meeting of the OIE Ad Hoc Group [13] Three “Other” options were included in this part of our survey, followed by an open-ended text box allowing respondents to freely specify any diseases not listed but deemed significant.
(iii) Pathogen-specific information. This part of the survey records basic information such as the stages of the outbreak, the incidence rate, and how it is being managed. Each question also offered an open-boxed “Other” option to enable respondents to provide any additional input.
(iv) Current vaccine perceptions and improvement expectations. This part of the survey explores the perceived efficacy of current vaccines and the challenges associated with their use. Respondents are prompted to evaluate vaccines based on factors such as effectiveness, cost, availability, and administration difficulties. It also required the identification of the drawbacks of the current vaccines. And what their expectations are for future vaccines. If respondents did not pick a vaccine as one of their answers to the previous question in part 3 regarding how they manage the disease, they will skip part 4 and turn to part 5 instead. Again, an open-boxed “Other” option was offered in each question.
(v) Expectations for future vaccines. This section aims to understand respondents’ opinions on future vaccines, gathering their expectations and acceptable thresholds for parameters such as effects on morbidity and mortality, and the associated cost of this ‘ideal vaccine.’ Additionally, respondents who did not complete the fourth section will be asked to specify the two key attributes they would most like to see in future vaccines.

### 2.4. Interview

At the end of the survey, respondents were asked whether they would be willing to participate in a 30-min online follow-up interview. They were asked to leave a convenient contact for further scheduling if agreed. The purpose of this interview was to provide an opportunity for respondents to share their insights on open-ended questions, offering perspectives beyond the scope of the predefined multiple-choice survey format. To ensure uniformity, we asked the respondents the following questions: 1. How do you perceive the relationship between vaccines and antibiotics in the industry today? 2. Which disease do you believe could potentially be eliminated first? What role can vaccines play in achieving this? 3. What types of support (e.g., financial, educational, regulatory) do you think are essential to facilitate the reduction in antimicrobial use?

### 2.5. Data Analysis and Statistical Methods

Only 100% completed surveys were considered valid and used for further analysis. Data and results were first exported from Qualtrics to Microsoft Excel for initial statistical management. We extracted and summarized data from the raw report generated using Microsoft Excel, and these data were then imported into GraphPad Prism (version 10) for further statistical analysis and visualization. Statistics, such as respondents’ ratings, stages of disease outbreaks, diagnostic methods, and treatment strategies, were generated into intuitive visual charts using GraphPad Prism 10. Two-tailed, unpaired *t*-tests were used to compare incidence rates and vaccine costs per dose between current and ideal vaccines at each stage. A 95% confidence interval (CI) was set for all conducted significance tests between groups. All significant results (*p* < 0.05) are indicated by an asterisk on the figures, while “ns” represents the comparison yielded a non-significant outcome. The figure for all respondents’ geographic distribution across the US was generated by Microsoft Power BI (Version 2.131.901.0). The rest of the illustrations were created by the authors using Adobe Illustrator 2020. All tables were generated by Microsoft Excel (Version 2509 Build 16.0.19231.20138).

A five-point Numeric Rating Scale (NRS) was adopted to assess multiple dimensions of vaccine evaluation, including effectiveness, cost, and availability. For effectiveness, a score of 1 indicated not effective at all and 5 indicated extremely effective. For cost, a score of 1 represented very cheap and 5 represented very expensive. For availability, a score of 1 indicated extremely difficult to obtain and 5 indicated extremely easy to obtain. To minimize potential misunderstanding of the scale, each numeric option was accompanied by descriptive text in the questionnaire. Responses were treated as ordinal data but summarized as mean values with 95% confidence intervals for comparative purposes. This structured NRS approach ensured consistency and comparability across respondents’ subjective assessments.

## 3. Results

This section is divided by subheadings. It aims to provide a concise and precise description of the experimental results, their interpretation, as well as the experimental conclusions that can be drawn.

### 3.1. Participant Recruitment and Demographics

**Figure 2:**
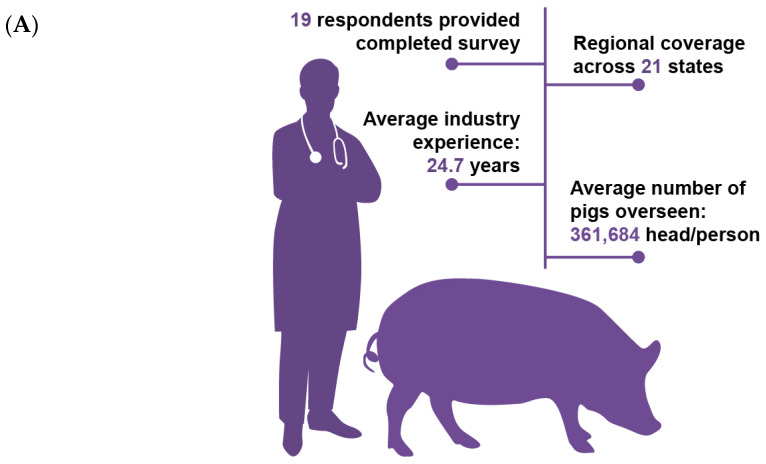

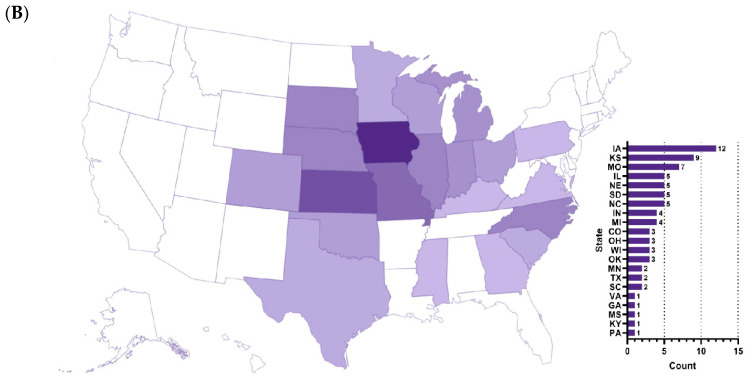
An overview of survey respondents’ (**A**) professional backgrounds and (**B**) geographical distribution.

Out of 202 potential participants contacted via email, 46 responded, resulting in a 22.8% response rate. Of these, 19 surveys were fully completed, yielding a 41.3% completion rate for returned surveys. Only these fully completed surveys were considered valid and used for further analysis. The valid respondents represented 21 different states and possessed an average of 24.7 years of industry experience. On average, each respondent was directly or indirectly responsible for managing 361,684 pigs at the time of the survey (Figure 2A). The geographical distribution of respondents closely mirrored that of the distribution of the swine population in the U.S., with the majority concentrated in central swine-producing states. Iowa (IA) had the highest number of respondents (Figure 2B).

### 3.2. Top Bacterial Diseases Requiring Improved Vaccines

**Figure 3:**
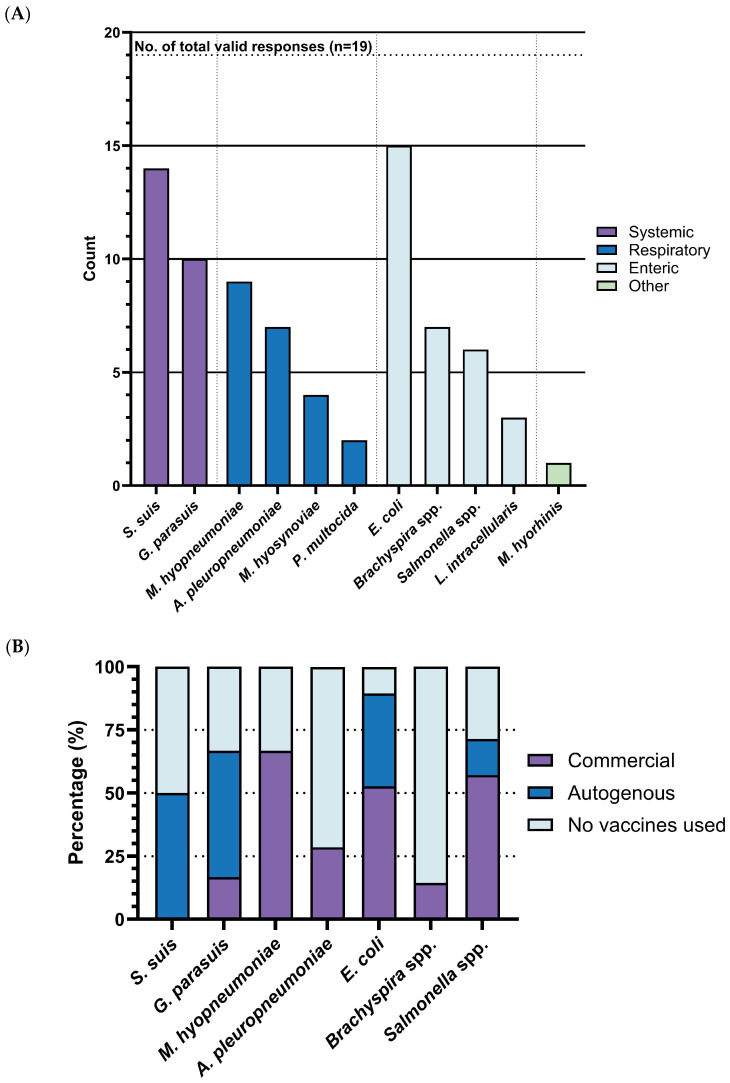

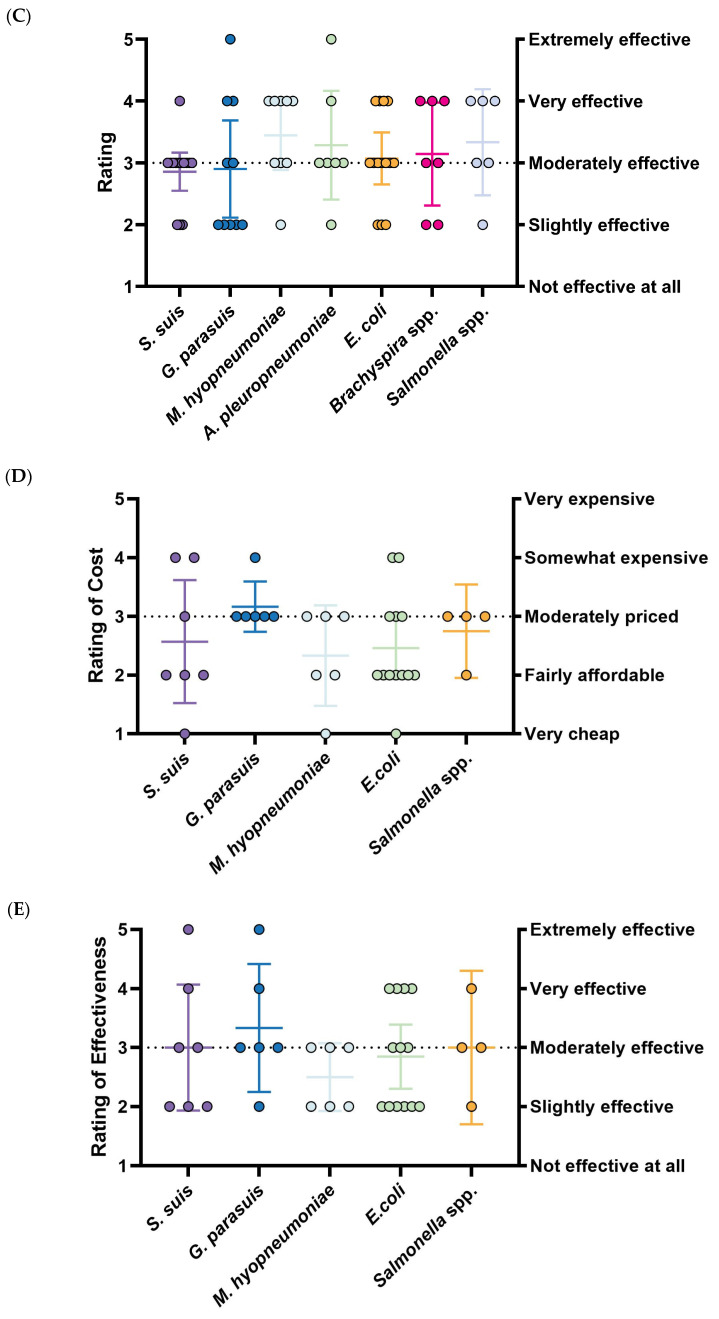

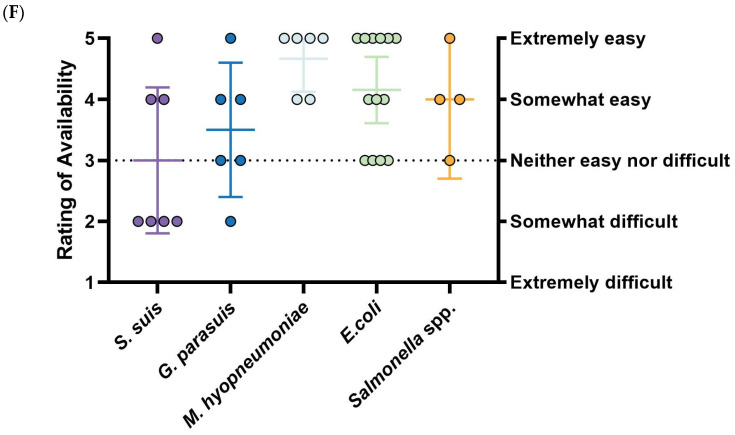
Horizontal comparison of key vaccine attributes across diseases. (**A**) Response counts for diseases demanding improved or new vaccines (total responses: *n* = 19). (**B**) Proportion of vaccine types in use (prescription vaccines included under autogenous vaccines). (**C**) Overall ratings of effectiveness for current management measures. (**D**) Ratings of current vaccines in terms of cost. (**E**) Ratings of current vaccines in terms of effectiveness. (**F**) Ratings of current vaccines in terms of availability. All dot plots display mean values and 95% confidence intervals (CI).

Our survey identified *Escherichia coli* (*E. coli*), *Streptococcus suis (S. suis)*, and *Glaesserella parasuis* (*G. parasuis*) as the top three bacterial diseases most urgently requiring improved vaccines. Over half of respondents selected these diseases, with rates of 78.9% (15/19), 73.7% (14/19), and 52.6% (10/19), respectively. These were followed by *Mycoplasma hyopneumoniae* (*M. hyo)* with rates of 47.4% (9/19), *Actinobacillus pleuropneumoniae* with rates of 36.8% (7/19), *Brachyspira* spp. with rates of 36.8% (7/19), and *Salmonella* spp. with rates of 31.6% (6/19). In contrast, *Mycoplasma hyosynoviae* with rates of 21.0% (4/19), *Lawsonia intracellularis* with rates of 15.8% (3/19), *Pasteurella multocida* with rates of 10.5% (2/19), and *Mycoplasma hyorhinis* with rates of 5.3% (1/19) were selected by only a small number of respondents (Figure 3A). Based on these response rates and statistical significance, our subsequent analysis was focused on the top four diseases: *E. coli*, *S. suis*, *G. parasuis*, and *M. hyo*. Notably, few respondents proposed additional bacterial diseases, further reinforcing the consensus around the importance of these identified bacterial diseases.

### 3.3. Vaccine Landscape, Expectations and Challenges for Urgent Bacterial Diseases in Swine

For the most urgently needed bacterial disease vaccines-*S. suis*, *G. parasuis* and *E. coli*, notably rely on autogenous vaccines, with usage rates of 50% (7/14), 50% (6/12), and 36.8% (7/19), respectively (Figure 3B). In contrast, the percentages of commercial vaccines utilized for these diseases are 0% (0/14), 16.67% (2/12), and 52.6% (10/19). The lower perceived effectiveness of management for these diseases (*S. suis*: 2.86, 95%CI: 2.55–3.17, *G. parasuis*: 2.90, 95%CI: 2.11–3.69, and *E. coli*: 3.07, 95%CI: 2.65–3.49) (Figure 3C) likely contributes to the higher dependence on autogenous options.

The effectiveness rating of *S. suis* vaccines is highly inconsistent with a wide distribution (3.00, 95%CI: 1.93–4.07). *S. suis* vaccines are considered the least accessible, with over half of respondents rating their availability as “somewhat difficult” (3.00, 95%CI: 1.80–4.19). *G. parasuis* vaccines are regarded as the most effective among prioritized diseases (3.33, 95%CI: 2.25–4.42) and reasonably accessible (3.50, 95%CI: 2.40–4.60), but they are also the most expensive (3.17, 95%CI: 2.74–3.60). *E. coli* vaccines are considered inexpensive (2.46, 95%CI: 1.93–2.99) and easy to obtain (4.15, 95%CI: 3.61–4.70), though their effectiveness is rated as below moderate (2.85, 95%CI: 2.30–3.39), mirroring trends seen with *M. hyo* vaccines. The results of *M. hyo* vaccines are quite distinct: they are exclusively commercial (66.6%, 6/9) and received the highest ratings for existing control measures (3.44, 95%CI: 2.89–4.00). While these vaccines are the cheapest (2.33, 95%CI: 1.48–3.19) and most accessible (4.67, 95%CI: 4.13–5.20), they are simultaneously rated as the least effective (2.50, 95%CI: 1.93–3.08) (Figure 3D–F). Due to limited responses and a lack of significance, *A. pleuropneumoniae*, *Brachyspira* spp., and *Salmonella* spp. were not analyzed further.

#### 3.3.1. *S. suis* in Swine Production: Current Landscape, Vaccine Expectations and Challenges

**Figure 4:**
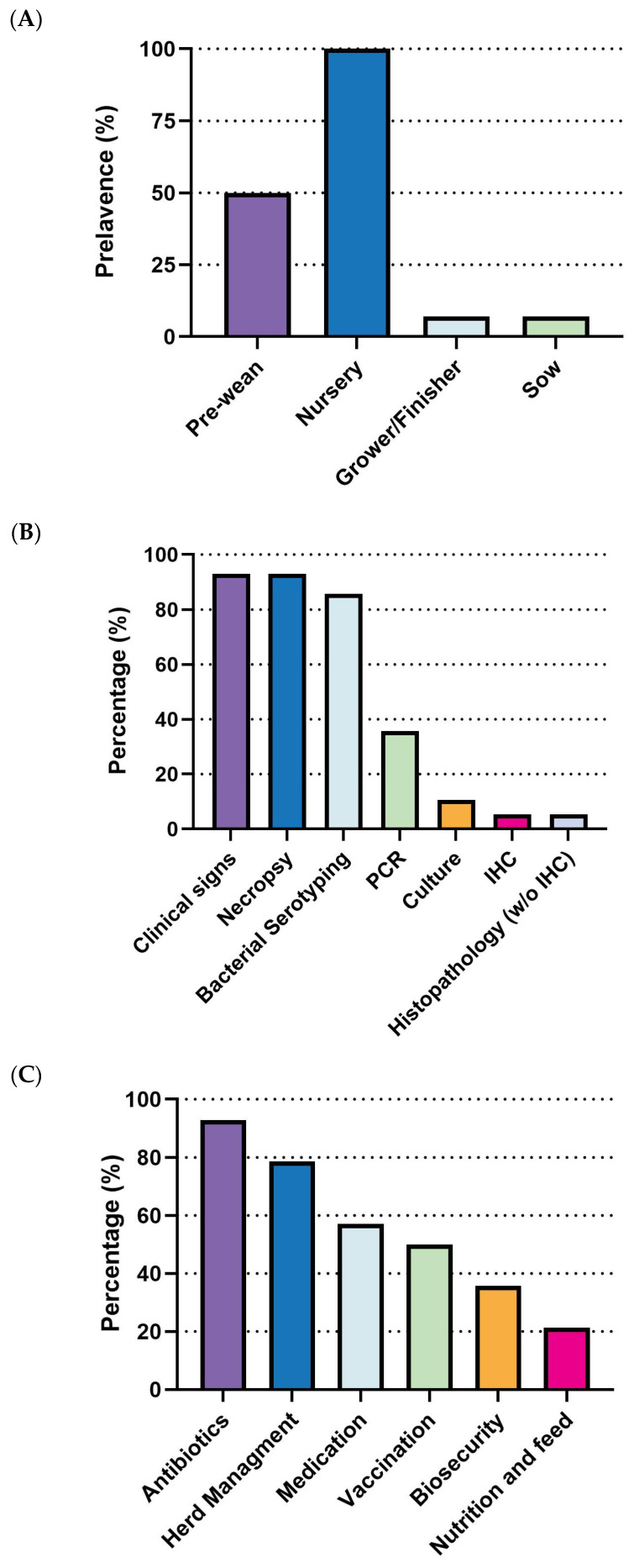
Summary of responses on *S. suis.* (**A**) Outbreak stages. (**B**) Diagnostic methods. (**C**) Management strategies. Number of responses: *n* = 14.

*S. suis* outbreaks primarily affect the early stages of swine production. All surveyed respondents (100%, 14/14) reported cases in the nursery stage, making it the highest incidence rate. The pre-weaning stage followed with a 50% incidence rate (7/14), while both grower/finisher and sow stages reported significantly lower rates (1/14) (Figure 4A). Diagnostic efforts largely rely on clinical signs and necropsy (92.8%, 13/14) and bacterial serotyping (85.7%, 12/14). Less frequent methods include PCR (35.7%, 5/14), culture, immunohistochemistry (IHC), and histopathology (without IHC) (Figure 4B). Regarding prevention and control strategies, there is a clear prioritization of antibiotics, with 92.9% (13/14) of respondents using them, followed by herd management (78.6%, 11/14). Medication (57.1%, 8/14) and vaccination (50.0%, 7/14) are moderately utilized, while biosecurity (35.7%, 5/14) and nutrition and feed (21.4%, 3/14) are the least adopted strategies (Figure 4C).

**Figure 5:**
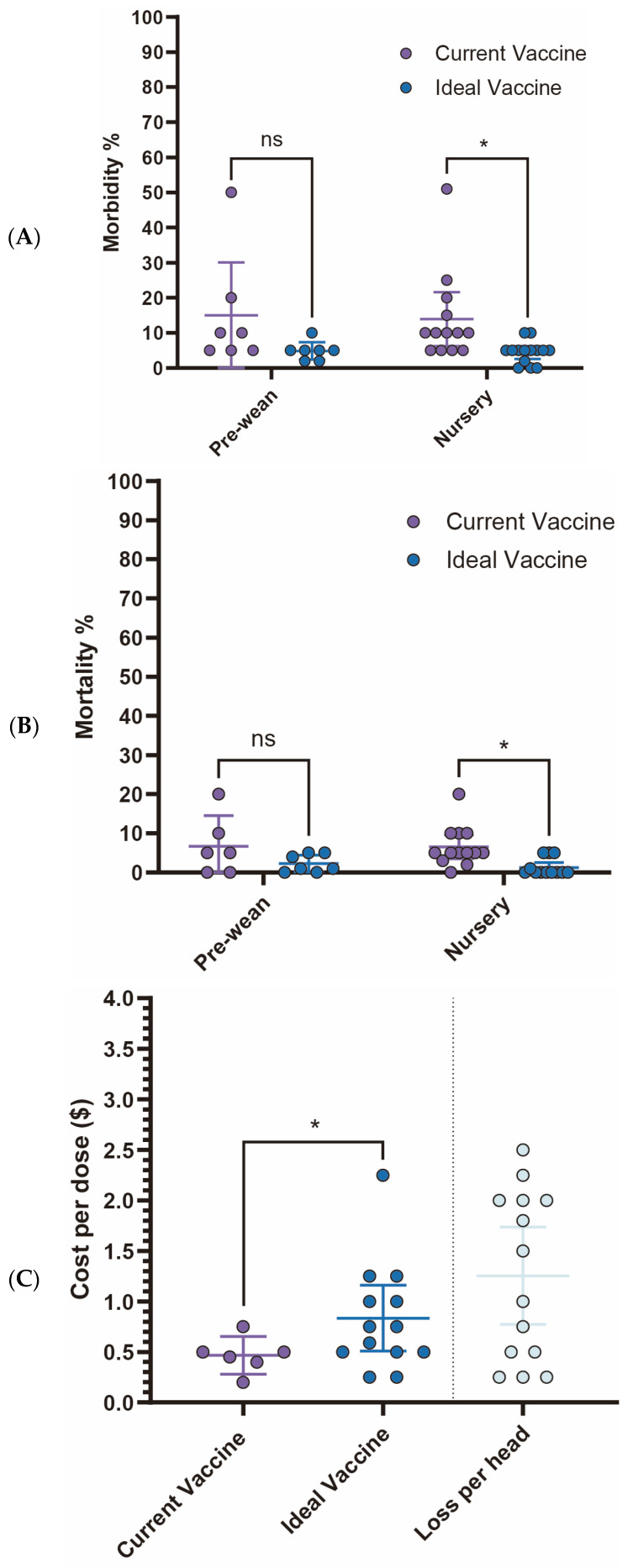
Comparison of key parameters between existing and ideal vaccines for *S. suis*. (**A**) Comparison of morbidity. (**B**) Comparison of mortality. (**C**) Comparison between the current vaccine price and the maximum price respondents are willing to pay for an ideal vaccine. Asterisks (*) indicate *p* < 0.05, while “ns” indicates “non-significant”.

Current *S. suis* vaccine fails to meet veterinarians’ expectations, particularly concerning morbidity and mortality rates in the nursery stage. While no statistically significant differences are observed in the pre-wean stage, the nursery stage showed a significant reduction in morbidity from 13.9% (95% CI: 6.26–21.60) with current vaccines to 4.4% (95% CI: 2.14–6.75) with an ideal vaccine (*p* < 0.05). Similarly, nursery mortality rates significantly declined from 6.5% (95% CI: 3.48–9.60) to 1.2% (95% CI: −0.07–2.54) (Figure 5A,B). Economically, current *S. suis* vaccines are priced at an average of $0.47 per dose (95% CI: $0.28–$0.65). However, survey participants expressed a willingness to pay an average of $0.83 per dose (95% CI: $0.51–$1.16) for an ideal vaccine that significantly reduces morbidity and mortality (Figure 5C, Table 1). With an estimated loss $1.254 per head (95% CI: $0.77–$1.74), this suggests a promising opportunity for a 1.786-fold increase in price for a more effective vaccine. The primary challenges associated with current *S. suis* vaccines, as summarized in Table 2, include limitations in efficacy: lack of cross-protection against new variants (85.7%) and broader-spectrum protection (71.4%), availability issues delay in vaccine distribution (71.4%), and adverse effects like local reactions (42.9%).

#### 3.3.2. *Escherichia coli* (*E. coli*) in Swine Production: Current Landscape, Vaccine Expectations and Challenges

**Figure 6:**
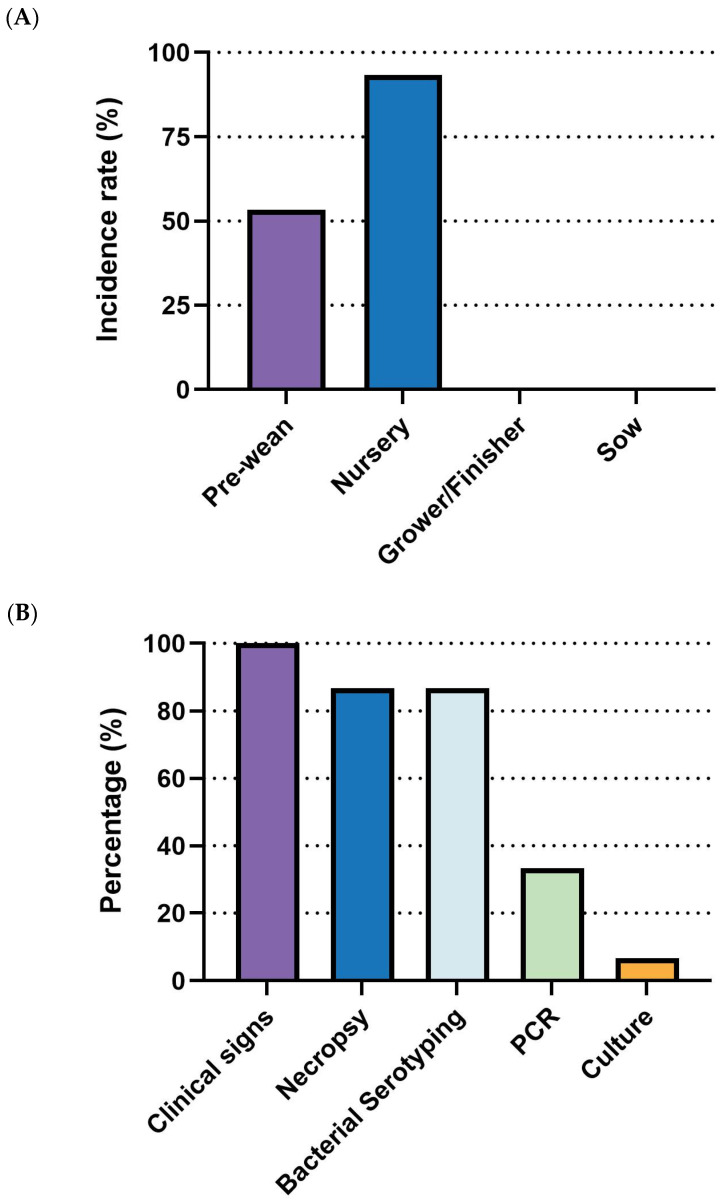

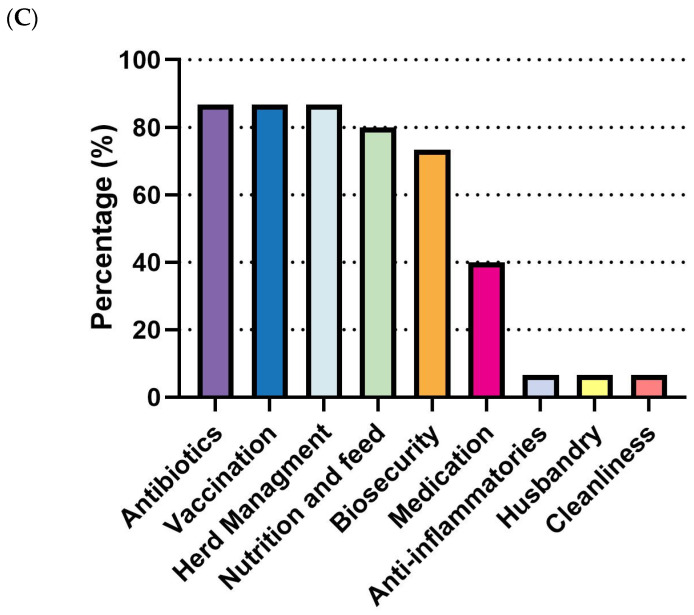
Summary of responses on *E. coli*. (**A**) Outbreak stages. (**B**) Diagnostic methods. (**C)** management strategies. Number of responses: *n* = 15.

*E. coli* outbreaks in swine production are most common in nursery-stage pigs, showing the highest incidence (93.3%, 14/15), followed by the pre-wean stage (53.3%, 8/15). The grower/finisher and sow stages were not reported as significant outbreak periods (Figure 6A). For diagnosis, clinical signs (100%, 15/15) were universally used for disease identification, followed by necropsy (86.7%, 13/15) and bacterial serotyping (86.7%, 13/15). PCR was moderately utilized (26.7%, 4/15), while culture was rarely employed (6.7%, 1/15) (Figure 6B). As for management, antibiotics (86.7%, 13/15), vaccination (86.7%, 13/15), and herd management (86.7%, 13/15) were the most commonly employed measures. Nutrition and feed interventions (80%, 12/15) and biosecurity (73.3%, 11/15) were also frequently adopted. Medication (40%, 6/15) and other measures, including anti-inflammatories, husbandry, and cleanliness (6.7%, 1/15 each), were less commonly reported (Figure 6C).

**Figure 7:**
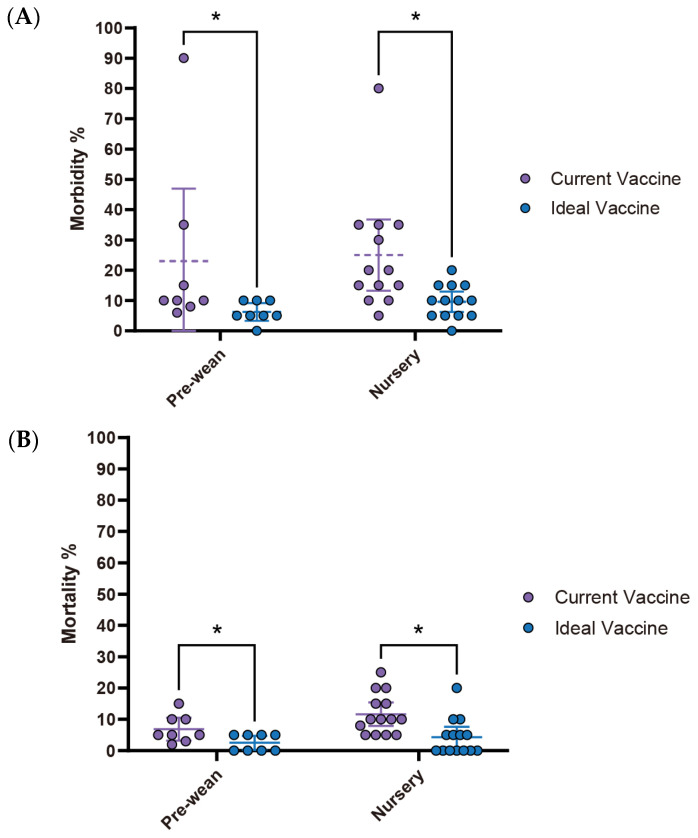

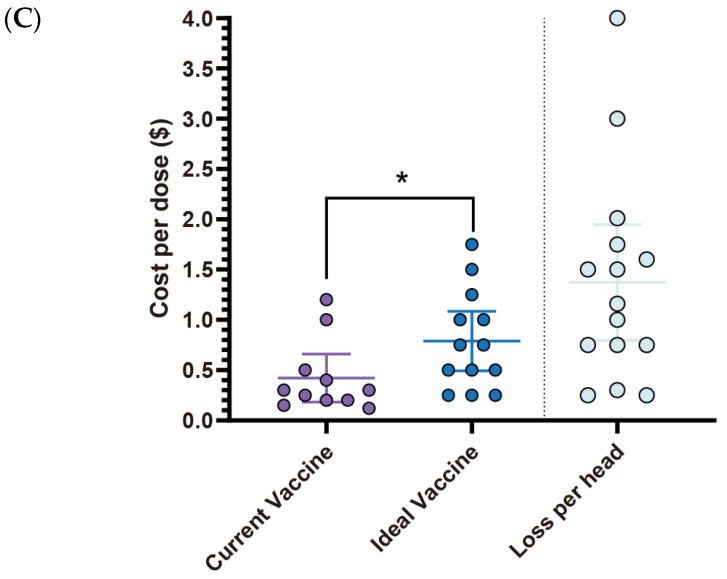
Comparison of key parameters between existing and ideal vaccines for *E. coli*. (**A**) Comparison of morbidity. (**B**) Comparison of mortality. (**C**) Comparison between the current vaccine price and the maximum price respondents are willing to pay for an ideal vaccine. Asterisks (*) indicate *p* < 0.05.

Respondents expressed significantly higher expectations for improvements in *E. coli* vaccines, noting substantial differences between current and ideal vaccine performance in both pre-wean and nursery stages. In the pre-wean stage, morbidity with an ideal vaccine is anticipated to decrease from 23% to 6.3%, while in the nursery stage, it’s expected to drop from 25% to 9.6%. Similarly, mortality rates are projected to fall from 6.9% to 2.5% in pre-wean piglets and from 11.6% to 4.2% in the nursery, highlighting the desired impact of improved vaccines (Figure 7A,B).

The average price of the current *E. coli* vaccine is $0.42 (95% CI: $0.18–$0.66), while the ideal vaccine price is able to reach $0.79 (95% CI: $0.49–$1.08), reflecting a fold change of 1.88. This perceived value aligns with the estimated loss of $1.37 (95% CI: $0.79–$1.95) per affected pig (Figure 7C, Table 3), suggesting a willingness to invest more for better protection. Current *E. coli* vaccines face several challenges (Table 4). The most frequently reported issue was the short duration of protection (54.5%), followed by lack of broader-spectrum protection, interference from maternal antibodies, and timing of administration, each reported by 36.4% of respondents. As for availability, difficulty in administration (45.5%) was the most significant concern, which is closely related to issues such as timing of administration and transient duration. Adverse effects are not a major concern (Table 4).

#### 3.3.3. *Mycoplasma hyopneumoniae* (*M. hyo*) in Swine Production: Current Landscape, Vaccine Expectations and Challenges

**Figure 8:**
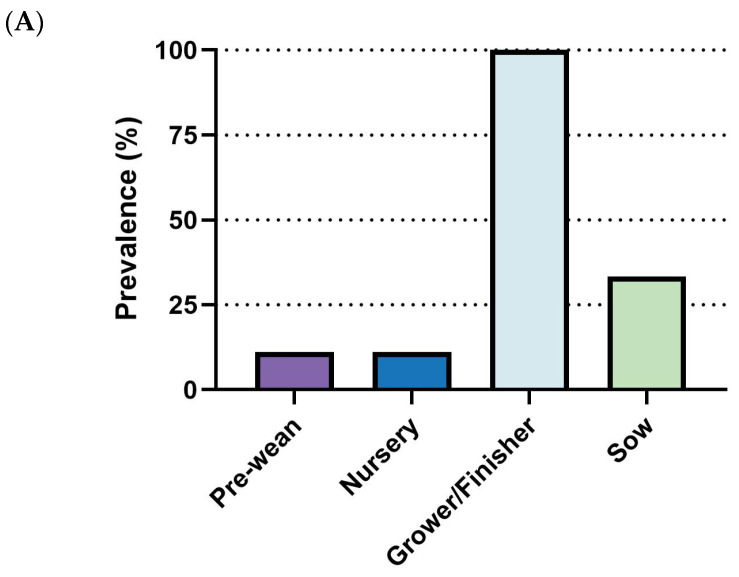

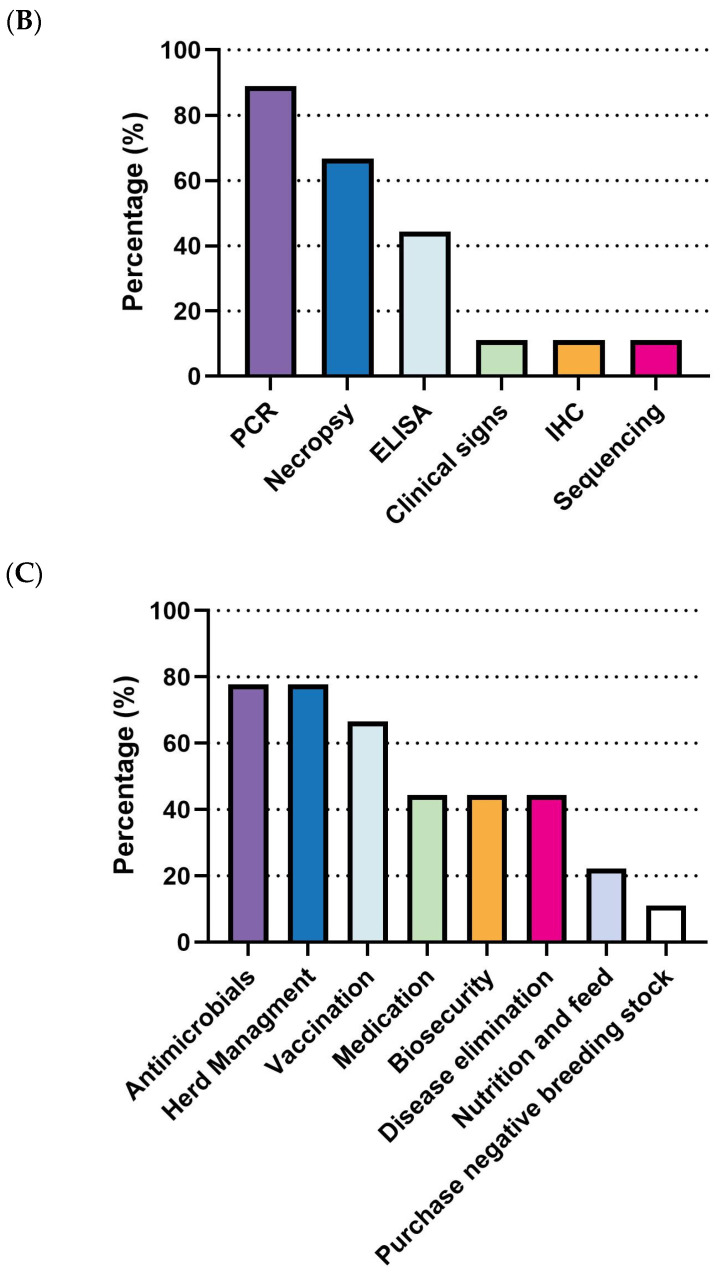
Summary of responses on *M. hyo.* (**A**) Outbreak stages. (**B**) Diagnostic methods. (**C**) management strategies. Number of responses: *n* = 9.

Outbreaks of *M. hyo* primarily affect the grower/finisher phase, with 100% (9/9) of respondents identifying it as the main stage for outbreaks. The sow stage followed with a lower prevalence rate (33.3%, 3/9), while pre-wean and nursery stages showed minimal reported incidence (Figure 8A). For diagnosis, PCR was the most frequently employed diagnostic tool (88.9%, 8/9), followed by necropsy (66.7%, 6/9) and ELISA (44.4%, 4/9). Other diagnostic approaches, including clinical signs, immunohistochemistry (IHC), and sequencing, were rarely utilized (11.1%, 1/9 each) (Figure 8B).

Regarding management strategies, antimicrobials and herd management were the most commonly adopted measures (77.8%, 7/9), followed by vaccination (66.7%, 6/9). Medication, biosecurity, and disease elimination each accounted for 44.4% (4/9). Nutrition and feed interventions and the purchase of negative breeding stock were reported at lower frequencies (Figure 8C).

**Figure 9:**
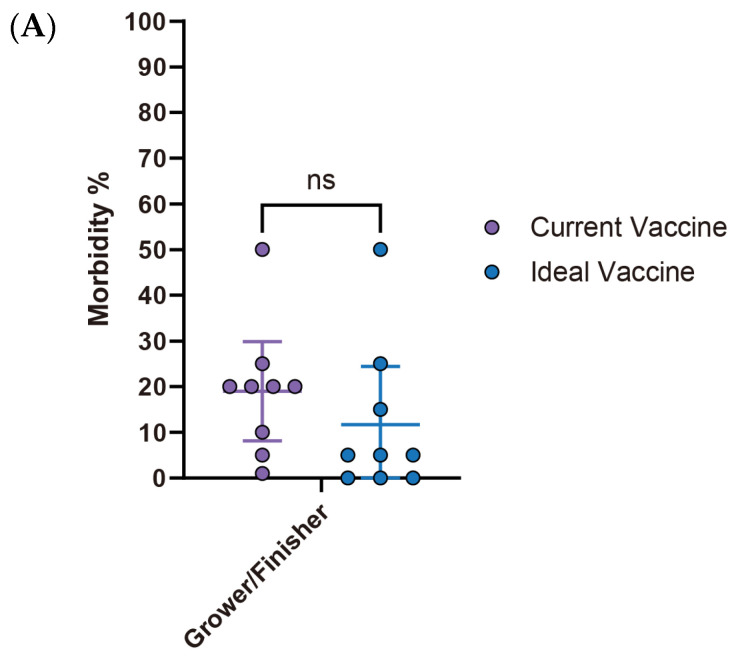

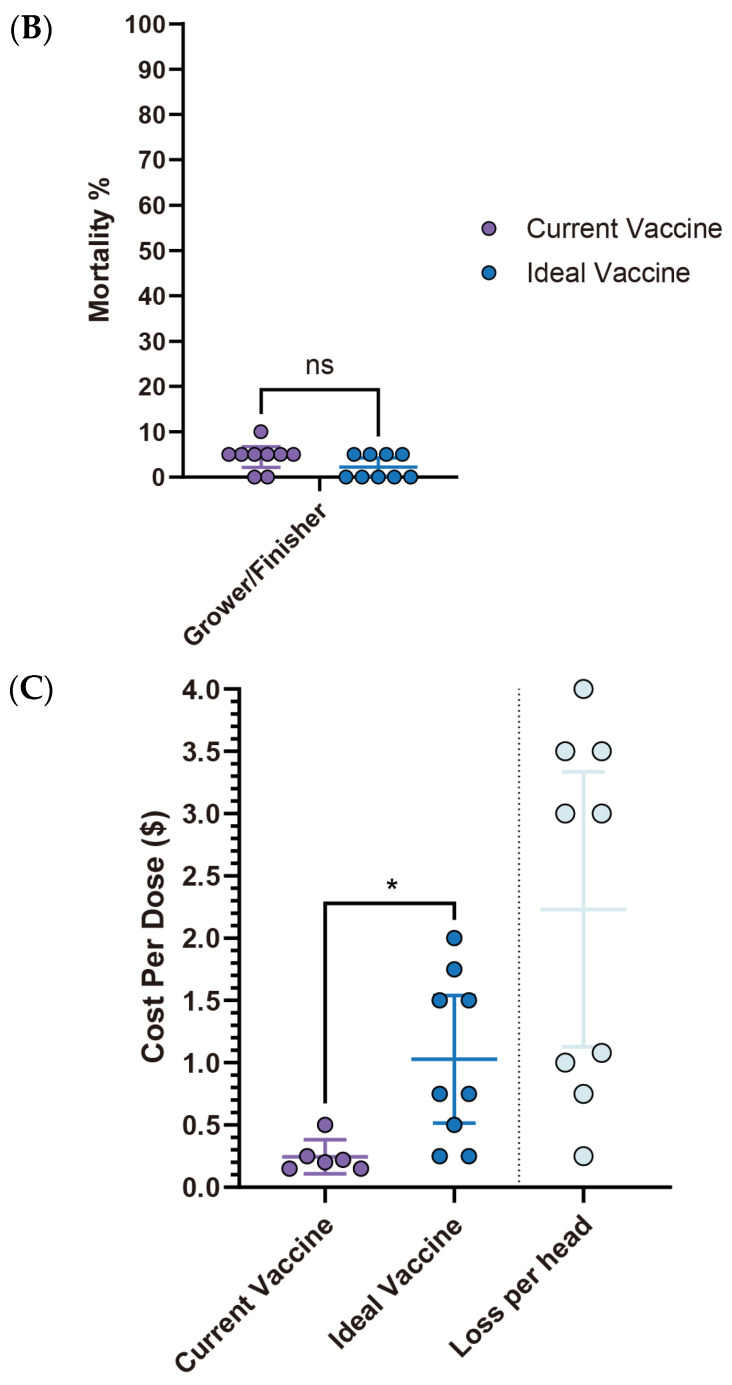
Comparison of key parameters between existing and ideal vaccines for *M. hyo*. (**A**) Comparison of morbidity. (**B**) Comparison of mortality. (**C**) Comparison between the current vaccine price and the maximum price respondents are willing to pay for an ideal vaccine. Asterisks (*) indicate *p* < 0.05, while “ns” indicates “non-significant”.

Interestingly, despite its high economic impact (estimated loss of $2.23 per affected pig), *M. hyo* vaccine improvements showed the lowest expectations among the four diseases analyzed. There were no significant differences (ns) observed in both morbidity and mortality rates between the current and ideal vaccines. Specifically, morbidity decreased from 19% (95% CI: 8.12–29.88) with the current vaccine to 11.7% (95% CI: −1.08–24.41) with the ideal vaccine, while mortality declined from 4.4% (95% CI: 2.14–6.75) to 2.2% (95% CI: 0.20–4.25), with neither showing statistical significance (Figure 9A,B). However, a notable observation lies in the vaccine price. The ideal vaccine’s price not only exhibited the most significant fold change (4.2 times) compared to the current vaccine, rising from $0.25 (95% CI: $0.11–$0.38) to $1.03 (95% CI: $0.52–$1.54), but also had the highest average cost per dose at $1.03 (95% CI: $0.52–$1.54). Moreover, the estimated economic loss per affected pig was also the highest among the four diseases, averaging $2.23 (95% CI: $1.13–$3.34) (Figure 9C, Table 5). The primary challenge with current *M. hyo* vaccines is their poor quality (66.7%, 4/6). This primary concern is accompanied by the short duration of protection (33.3%, 2/6) and shortage of vaccine storage (33.3%, 2/6). Adverse effects are not a major concern (Table 6).

#### 3.3.4. *Glaesserella parasuis* (*G. parasuis*) in Swine Production: Current Landscape, Vaccine Expectations and Challenges

**Figure 10:**
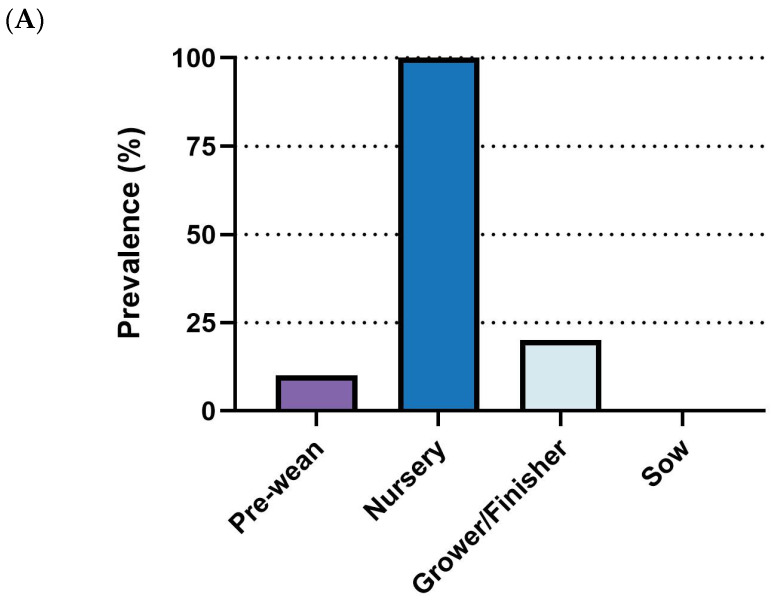

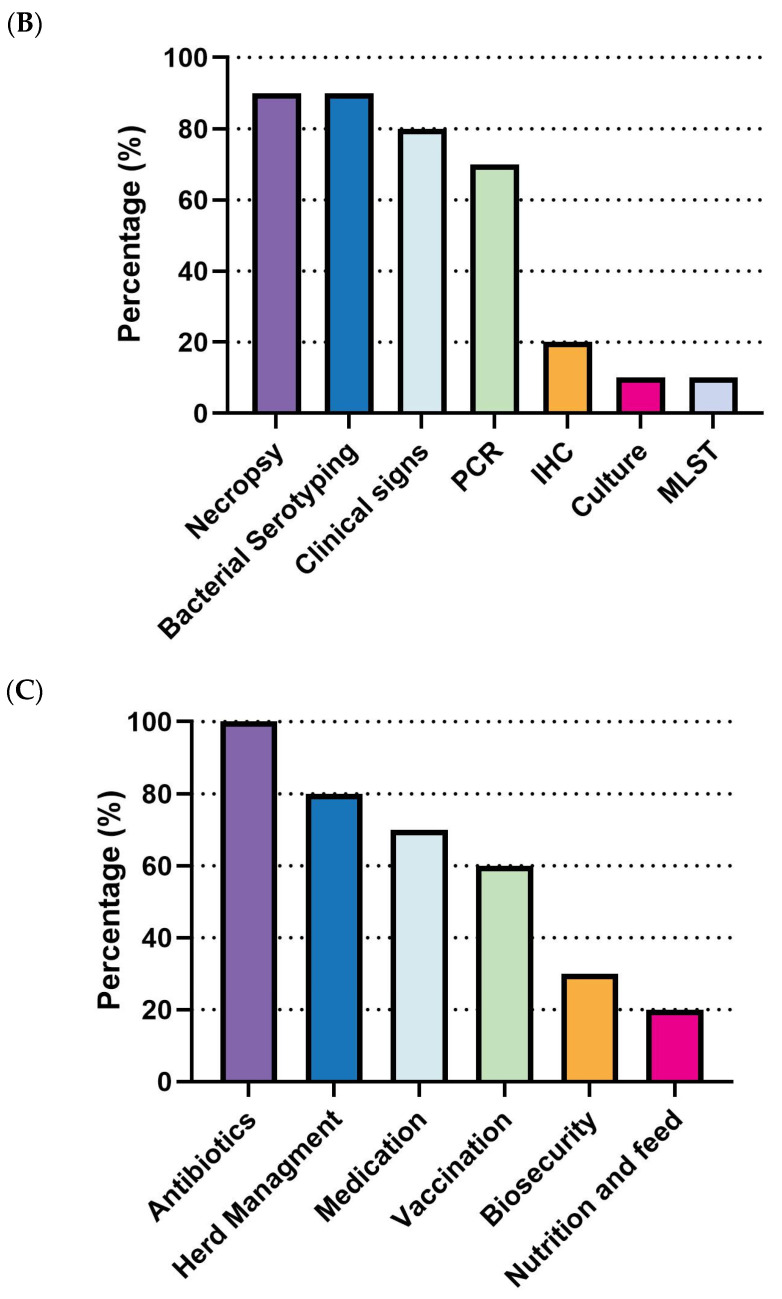
Summary of responses on *G. parasuis*. (**A**) Outbreak stages. (**B**) Diagnostic methods. (**C**) Management strategies. Number of responses: *n* = 10.

*G. parasuis* outbreaks are most prevalent in the nursery stage, with all respondents (100%, 10/10) identifying it as the primary phase for disease occurrence. Other stages showed significantly lower prevalence, remaining under 20% (Figure 10A). For diagnostic methods, necropsy (90%, 9/10), bacterial serotyping (90%, 9/10), clinical signs (80%, 8/10), and PCR (70%, 7/10) were the most commonly employed approaches. Culture (10%, 1/10), immunohistochemistry (IHC, 20%, 2/10), and Multi-Locus Sequence Typing (MLST) were rarely applied (Figure 10B). Regarding management strategies, antibiotics were universally used by all respondents (100%, 10/10), followed by herd management (80%, 8/10) and medication (70%, 7/10). Vaccination was employed by 60% (6/10) of the respondents. Lastly, biosecurity (30%, 3/10) and nutrition/feed strategies (20%, 2/10) were less frequently adopted (Figure 10C).

**Figure 11:**
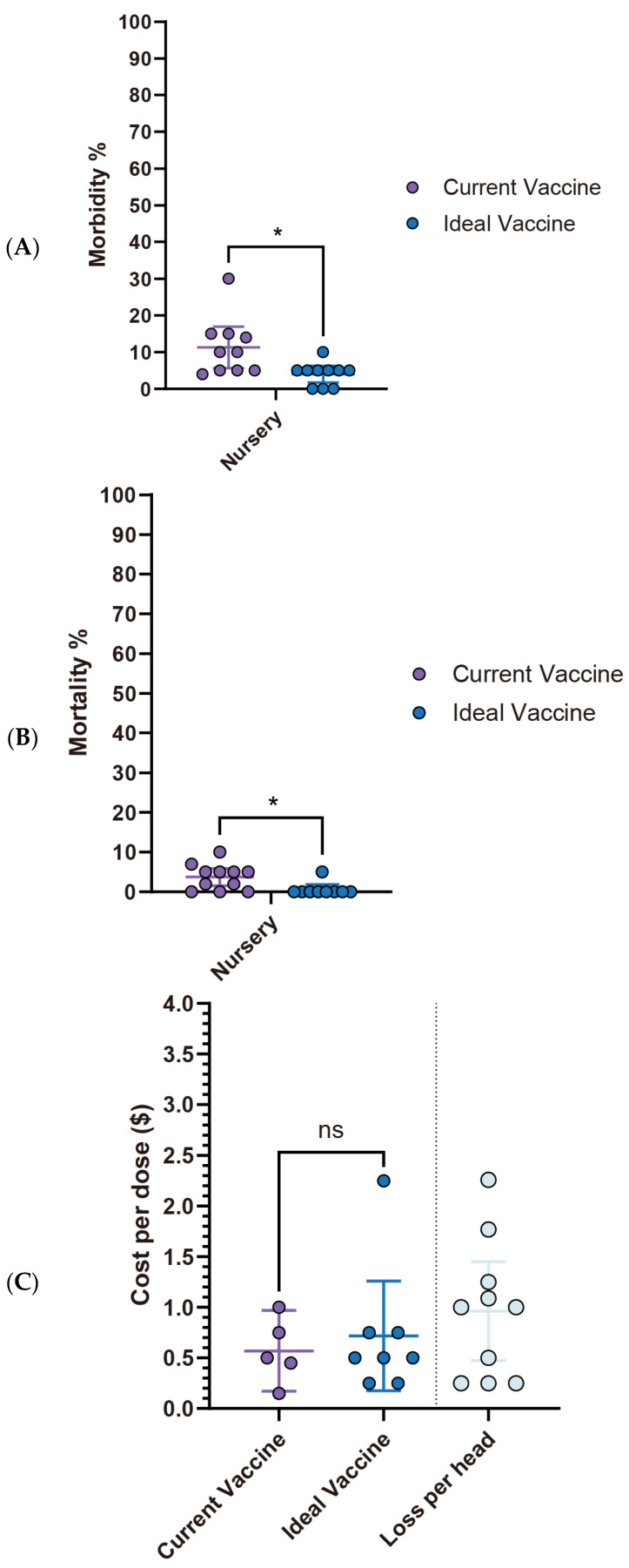
Comparison of key parameters between existing and ideal vaccines for *G. parasuis*. (**A**) Comparison of morbidity. (**B**) Comparison of mortality. (**C**) Comparison between the current vaccine price and the maximum price respondents are willing to pay for an ideal vaccine. Asterisks (*) indicate *p* < 0.05, while “ns” indicates “non-significant”.

The gap between current and ideal *G. parasuis* vaccines remains significant. In the nursery stage, an ideal vaccine is projected to reduce morbidity from 11.3% (95% CI: 5.66–16.94) to 4.0% (95% CI: 1.74–6.26) and mortality from 4.1% (95% CI: 1.85–6.35) to 0.5% (95% CI: −0.63–1.63), demonstrating substantial expected improvements in efficacy (Figure 11A,B). The estimated economic loss per affected pig was approximately $0.96 (95% CI: $0.47–$1.45), while the cost per dose, the mean price of the current vaccine, was $0.57 (95% CI: $0.17–$0.97), and respondents only willing to pay a price slightly higher than that for an ideal vaccine, at $0.72 (95% CI: $0.18–$1.26). This suggests that while improvements are desired, there’s a limited acceptable price increase for enhanced performance (Figure 11C, Table 7). Current *G. parasuis* vaccines do not exhibit particularly prominent shortcomings (Table 8), but key concerns include a lack of cross-protection against new variants (33.3%), limited broader-spectrum protection (33.3%), and efficacy interference by maternal antibodies (33.3%). Additionally, delays in vaccine availability (33.3%) and high costs (33.3%) were noted as issues, alongside reported adverse effects like local reactions (50.0%) and temporary decreases in productivity (33.3%) (Table 8).

### 3.4. Interview Insights

We conducted interviews with three highly experienced veterinarians from the respondent pool, each possessing an average of 35.3 years of professional experience. Their work (or past roles) spans academic institutions, industry, and associations. Hereafter, they will be referred to as Dr. A, Dr. B, and Dr. C.

#### 3.4.1. Relationship Between Vaccines and Antibiotics

When asked about the relationship between vaccines and antibiotics, the veterinarians shared varied and thoughtful insights.

Insights from Dr. A: *“It’s a matter of do vaccines give us an opportunity of more effectively, more judiciously use of our antimicrobials, but we are never going to completely eliminate the use of antimicrobials. There are some animals that don’t respond to the vaccine or some difficulties in administering it. Certainly, could make great opportunities to use less of it, right? Certainly, could make great opportunities to make them more effective when we do use them. For example, we know when we have PRRS-infected pigs that change the pharmacokinetics of antimicrobials. So, I think they are an additional tool, and every additional tool we get, the more judicious we can use the one we have available.”*

Insights from Dr. B: *“Maybe there are situations when vaccines would be an alternative. But generally, when I think about that, it would be an alternative to mass treatments. What we want to do is to decrease the routine use of feed-grade antibiotics in water, soluble antibiotics. But still, because of animal care, we want to be able to still do individual animal treatments. A great example is Streptococcus suis. If you look at the amount of Amoxicillin that is administered in the water across our entire system, it is quite high. Because it is relatively low-cost and the only alternative we have at this time. So there, if we had an effective vaccine, I would anticipate we would not have to use the Amoxicillin, but we still would use one of the injectables for the individual pig. So that’s kind of a good way to think about it.”*

Insights from Dr. C: *“I’m not sure about the term ‘alternative’. I don’t think it’s vaccines or antibiotics. I think there will always be a balance of the use of both. If we had an effective PRRS vaccine, as an example. Antibiotic use across the industry would go down for sure. But I don’t see vaccines replacing antibiotics entirely. See the balance changing.”*

#### 3.4.2. Potential for Disease Elimination

For the question “Which disease do you believe we can potentially eliminate first?”, the veterinarians highlighted those that compromise the pig’s immune system or have well-defined targets.

Insights from Dr. A: *“Well, I think by far the most important would be the primary infectious agents that would compromise the pigs’ immune system. Because that creates more opportunities for bacteria, so PRRS is definitely on that list, right? Mycoplasma hyopneumoniae would be on that list because they would impact the mucociliary defense to protect the lung. Influenza would certainly be on that list. Circovirus type 2, although we have a reasonably effective vaccine, there is always room for improvement on that one. I think any time we could take one element out of the equation, we start to skew the odds in favor of the pigs. And that’s what we really want to do. So, yeah, any of those diseases would simplify the picture, and the reason I say those diseases have an impact on the immune system is because I think then we will get a lot of secondary benefits, right? A lot of bacteria that kill pigs are just opportunistic because the pigs’ defenses are down. So if the pigs’ defenses are re-established or protected, then, you know, potentially will have an entirely different outcome. And we narrow the list of things they are going to be susceptible to.”*

Insights from Dr. B: *“Mycoplasma hyopneumoniae, we’re in the industry being very successful. Or if we had a more effective vaccine, you can look at it the other way. The Mycoplasma hyopneumoniae vaccine only cost us about 10 cents now a pig, a dose, but they’re not very effective. So, if there was technology that would give us a more effective vaccine. You would answer the question differently. Certainly, we know what PIC (Pig Improvement Company, a major global swine genetics company) did, they were able to eliminate Streptococcus suis, Actinobacillus suis and Mycoplasma hyorhinis. Now, you can’t do that on a commercial farm. But it still says the opportunities there to eliminate those bacteria. And we can successfully eliminate swine influenza today in the sow herds. We do it all the time. It takes us about six weeks. But the problem is they become reinfected, different strain comes in and affect the pig flow. We can eliminate it from the sow herds. So the pigs are negative at weaning to the virus, but most of them get contaminated in the growing period.”*

Insights from Dr. C: *“Yes. For example, M.hyo causes pneumonia and slow growth. We don’t deal with single infections a lot. Focused elimination is a good choice. All the pathogens alone, you know, Mycoplasma alone in a naive herd is very problematic. Mycoplasma alone and endemically infected herd as a drag on production. But we don’t see an increase in mortality now. We just see pneumonia and slow growth. More and more successful elimination of mycoplasma. So I’m not sure focusing on that one. As you look at viruses, especially the viruses in that complex situation, PRRS virus, influenza, our influence is difficult. We have vaccines that do an okay job reducing disease, but they’re very specific, and it’s a constantly changing virus, as is PRRS. So yeah, our commercial vaccines are never a perfect match for these viral diseases. So right, those are probably the bigger opportunities in my mind and where we’re lacking good products today. I would say influenza, PRRS, enteric viruses, PEDV, Rotavirus. Then on the bacterial side, Streptococcus suis. I mean, I have friends and colleagues working on that for the last three decades, and we still don’t have a great option for Streptococcus suis.”*

#### 3.4.3. Support Necessary for Antimicrobial Reduction

For the question “What kind of support (e.g., financial, educational, regulatory) do you think is necessary to facilitate the reduction in the use of antimicrobials.”, the veterinarians pointed to a mix of education, effective vaccines, and regulatory measures.

Insights from Dr. A: *“I think a much better understanding about antimicrobial stewardship, and how things contribute to stewardship. I think farms are going to be expected in the future to establish formal stewardship plans. Most of human health in U.S. has required to have a formal stewardship plan. Based on the core 5 principle that the AVMA has adopted to vet med in order to get their federal funding, we don’t have that sort of reward on veterinarians’ side. The owner pays the cost. So, I think we can expect some sort of regulatory legislation to force that issue. So, the sooner farms start thinking about that, the better.”*

Insights from Dr. B: *“It is more effective vaccines against these primarily bacterial pathogens and viral pathogens. If we’re Mycoplasma negative and we’re PRRS negative, our growing pigs do not have any antibiotics other than an individual pig treatment, and we would have mortality in, say, on the top 10% between 3 and 3.5%. And that mortality would be just for a number of reasons: lame, fighting. So the goal is that is to raise pigs without antibiotics. To do that, we’ve still got some gaps in our vaccines. And a big thing to put on your list would be, that still frustrates me, even in our autogenous and prescription vaccines, it tends to take us 12 to 14 weeks from the time we start to get a product. And I argue with our vets because in production, for 14 weeks is almost a third of a year. So, I have to tell my producer and myself, well, that’s, we’ll have something in four months. And yeah, anything you can do to shorten that would improve the opportunity for market.”*

Insights from Dr. C: *“Antibiotic use. Education is always important. Within the industry, we’re constantly monitoring antimicrobials resistance target by antibiotics stewardship. So I think the education is there and hopefully the industry, we’re trending with less and less antibiotic use. From a regulatory perspective, we’re limited in our antibiotic choices today. Hopefully don’t foresee more stringent regulatory use in antibiotics. We’re fairly limited in what we have to use today for animal welfare treatment.”*

## 4. Discussion

### 4.1. Prioritizing Vaccines for AMR Reduction in Swine Production

Vaccines are a proactive and essential strategy in modern swine production to minimize the reliance on antibiotics, thereby playing a vital role in curbing the global AMR threat. However, reports often fall short in identifying urgent demands, regional prevalence, or the practical feasibility of vaccine implementation in real-world swine production. Such as the OIE’s primarily provides a broad and general overview of various pathogens without identifying the most pressing demands [13]. To address this gap and prioritize diseases more effectively, we conducted this survey which revealed that U.S. swine veterinarians consistently identified *S. suis*, *E. coli*, *G. parasuis*, and *M. hyo* as the most urgent and impactful bacterial diseases requiring improved vaccines in swine production in the United States (Figure 3A). These pathogens bring significant and costly challenges to swine production, driving substantial antibiotic use and increasing concerns about AMR.

(i) *S. suis* is considered extremely difficult to either control or eradicate [23,24]. It significantly challenges early-life pigs, contributing to an estimated average loss of $1.25 per pig in this study. In European contexts, costs vary from €0.60 ($0.63) to €1.30 ($1.36) per pig [25]. While advanced diagnostics like whole-genome sequencing (WGS) and AI-driven predictive modeling could revolutionize surveillance [26,27], current management relies heavily on reactive antibiotic use due to limited vaccine efficacy and the pathogen’s high diversity [23,24]. Dr. B mentioned in the interview that expenditures on antimicrobials, particularly the use of beta-lactams (e.g., *Amoxicillin*) administered via the drinking water system, are substantial. Producers are willing to invest more in effective vaccines, emphasizing the need for both long-term cross-protective solutions and rapid serotype identification.
(ii) *E. coli* is a widespread pathogen with substantial economic impact. The majority of economic losses caused by *E. coli* stem from mortality, decreased weight gain, and expenses related to treatments, vaccinations, and supportive care associated with PWD (post-weaning diarrhea), ED (edema disease), and neonatal diarrhea [28,29,30]. Veterinarians estimated an average economic loss of $1.37 per pig in the US (Table 3). For comparison, European data estimate the cost per sow per year at €40 ($41.3) to €134 ($138.2), with a neonatal mortality rate of 10% and a post-weaning mortality rate of 1.5% [31]. Dr. B mentioned in the interview that he sometimes spends approximately $1.36 per pig to control post-weaning *E. coli* infections, a cost he deemed unsustainable. Although antibiotics remain the common strategy, it is currently viewed as a necessary but less-than-ideal solution due to the raising concern of AMR. For instance, the prevalence of multidrug resistance enterotoxigenic *E. coli* in swine production has been well-documented for more than a decade [30,32]. Those highlight the urgent need for better *E. coli* vaccine solutions, even if it means higher costs.
(iii) *M. hyo* is widely distributed worldwide and recognized as one of the most economically significant diseases affecting pig production [33]. It colonizes the ciliated epithelial cells of the respiratory tract, compromising both innate and adaptive pulmonary immunity, thereby substantially elevating the risk of secondary infections [34,35]. In cases of solely *M. hyo* infection, the estimated loss per pig is approximately $0.63. However, when co-infected with PRRSV or SIV, the economic losses rise significantly to $9.69 and $10.12 per pig, respectively [36]. In a naïve population, an outbreak is estimated to result in an average loss of $7.92 per pig [37]. Eradication is a key strategy. Studies have demonstrated the significant economic benefits of *M. hyo* eradication in swine production, with an estimated annual benefit of $877,375 per farm, or approximately $7.00 per pig marketed [38]. Current vaccines provide only incomplete protection, fail to prevent colonization, and do not significantly reduce the transmissibility of the pathogen [33]. In our findings, the efficacy rating of *M. hyo* vaccines ranked the lowest (Figure 3D). The economic burden and reliance on antibiotics for treating secondary infections drive the demand for new and improved vaccines. Under the well-established eradication protocols, there remains a strong willingness to invest significantly in optimized vaccines, even if the expected performance improvements are modest. The willingness to invest in enhanced vaccine solutions is evident, with the average price per dose increasing from $0.25 to $1.03, representing a 4.2-fold increase (Table 5). This makes the development of enhanced *M. hyo* vaccines one of the most promising investments, offering substantial economic benefits to both the pork industry and vaccine producers.
(iv) Compared to the above three pathogens analyzed, *G. parasuis* exhibits the lowest morbidity, mortality, and estimated economic impact (Figure 11A,B, Table 7). Veterinarians show limited willingness to significantly increase vaccination budgets, as current vaccine prices are near their perceived acceptable threshold (Table 7). Future *G. parasuis* vaccines must therefore prioritize cost control alongside improved efficacy, particularly in overcoming challenges such as unclear immunogenic processes, inconsistent protection across serovars, and the prevalence of non-typeable strains [39,40,41].

### 4.2. Balancing Science, Economics, and Real-World Challenges

Tackling AMR requires a multifaceted approach that extends beyond simply curbing antibiotic use or developing a vaccine. The successful development and deployment of new vaccines require a delicate balance of scientific innovation, economic viability, and real-world implementation. A successful vaccine must not only be effective against a specific pathogen but also be economically feasible to produce, and its adoption must be supported by sound policies, robust educational initiatives, and collaborative efforts across the industry. The cost of vaccine development involves substantial investments in funding, intellectual resources, and time, with some vaccines requiring decades of research without a guarantee of success. Just as farmers must constantly weigh costs against benefits, the decision to invest in vaccine development depends heavily on its potential return on investment (ROI), which considers technical hurdles, financial challenges, and market demand.

On the demand side, a vaccine’s value is determined by a variety of factors, including the pathogen’s geographic prevalence, the scale of economic losses it causes, and the availability of alternative solutions. For example, while pathogens like *S. suis* and *E. coli* have immense market potential, developing vaccines for them is unpredictable and expensive due to research obstacles. As Dr. C noted: “*I have friends and colleagues working on that for the last three decades, and we still don’t have a great option for S. suis.*”. This illustrates why vaccines for some of the most critical diseases remain elusive. Currently, large-scale antibiotic use remains the primary control measure, with limited alternative tools available. The following table (Table 9) summarizes the key factors influencing vaccine development for the top four bacterial diseases prioritized in the U.S., highlighting the delicate balance required.

Regulatory shifts, such as introducing prescription vaccines, have already proven effective by enabling a faster response to disease outbreaks. Additionally, government funding can make it feasible to develop vaccines for diseases with low prevalence or minimal economic incentives. On the educational front, it’s crucial to help farmers, especially those on independent farms, understand the tangible business benefits of adopting antimicrobial stewardship plans. When farmers view these practices not as an added burden but as a valuable tool for improving profitability and gaining a competitive edge, they are more likely to participate.

It is also important to address the public’s perception of AMR. While the public often worries that “contaminated” meat contributes directly to AMR [42], established food safety measures like pasteurization and cooking guidelines make the risk of AMR spreading through food products minimal. The primary driver of AMR in humans is the direct use of antibiotics in human medicine [43,44]. Therefore, a more effective strategy for mitigation in animal agriculture should focus on reducing bacterial shedding and improving environmental decontamination [45,46]. While a moderate reduction in antibiotic use is beneficial, a 100% antibiotic-free system may not be scientifically or economically justified, as it can lead to increased morbidity and higher production costs [47]. Transparent communication with the public about these complexities is key to balancing production efficiency with consumer expectations.

## 5. Conclusions

This study highlights the multifaceted challenges and opportunities associated with key bacterial vaccines as alternatives to antimicrobials in swine health management. We gathered insights from practicing swine veterinarians in the U.S. regarding the most critical vaccines needed for production, the limitations of current vaccines, and their expectations for future options. Based on this information, we evaluate the current landscape of disease prevention and control for various bacterial pathogens, identify the key factors influencing vaccine urgency, and suggest solutions tailored to production requirements. In addition to the academic challenges of vaccine development, this study also addresses economic, policy, and educational factors essential for effectively implementing vaccines as alternatives to antibiotics. We aim to provide a comprehensive discussion on the potential for bacterial vaccines to replace antibiotics in swine production, presenting future research directions and aiding in decision-making to effectively reduce AMR in the swine industry.

## Figures and Tables

**Table 1:** Comparison of economic parameters between current and ideal *S. suis* vaccines.

Data	n	Median	Mean	95%CI	Min–Max	SD	SEM
Estimated Loss, $/hd	14	1.250	1.254	0.77–1.74	0.25–2.5	0.835	0.223
Current Vaccines’ Price, $/hd	6	0.475	0.467	0.28–0.65	0.2–0.75	0.178	0.073
Ideal Vaccines’ Price, $/hd	13	0.750	0.834	0.51–1.16	0.25–2.25	0.540	0.150
Ideal price − current price, $/hd	-	0.275	0.367	-	-	-	-
Fold change	-	1.579	1.786	-	-	-	-

Note: “n”: number of responses; “-”: not available, “hd”: head.

**Table 2:** Key challenges and priorities for future *S. suis* vaccines.

Categories	n	Specific Issues	Percentage
Limitations	7	1. Lack of cross-protection against new variants	85.7% (6/7)
		2. Lack of a broader spectrum of protection	71.4% (5/7)
		3. Efficacy affected by maternal antibodies	57.1% (4/7)
Availability	7	1. Delays in vaccine distribution	71.4% (5/7)
		2. High cost	14.3% (1/7)
		3. Shortage of storage	14.3% (1/7)
Adverse effects	7	1. Local reactions	42.9% (3/7)
		2. Systemic reactions	28.6% (2/7)
		3. Allergic reactions	14.3% (1/7)
		4. Temporary decrease in productivity	14.3% (1/7)

Note: “n”: number of responses.

**Table 3:** Comparison of economic parameters between current and ideal *E. coli* vaccines.

Data	n	Median	Mean	95%CI	Min–Max	SD	SEM
Estimated Loss, $/hd	15	1.160	1.371	0.79–1.95	0.25–4	1.042	0.269
Current Vaccines’ Price, $/hd	11	0.300	0.420	0.18–0.66	0.12–1.2	0.356	0.107
Ideal Vaccines’ Price, $/hd	13	0.750	0.789	0.49–1.08	0.25–1.75	0.488	0.135
Ideal price- current price, $/hd	-	0.450	0.369		-	-	-
Fold change	-	2.500	1.879		-	-	-

Note: “n”: number of responses; “-”: not available, “hd”: head.

**Table 4:** Key challenges and priorities for future *E. coli* vaccines.

Categories	n	Specific Issues	Percentage
Limitations	11	1. Short duration of protection	54.5% (6/11)
		2. Lack of a broader spectrum of protection (e.g., Combination)	36.4% (4/11)
		3. Efficacy affected by maternal antibodies	36.4% (4/11)
		4. Timing of administration	36.4% (4/11)
Availability	11	1. Difficulty in administration	45.5% (5/11)
		2. High cost	9.1% (1/11)
		3. Strict storage requirement	9.1% (1/11)
Adverse effects	11	1. Systemic reactions	18.2% (2/11)
		2. Local reactions	9.1% (1/11)
		3. Risk of reversion to virulence	9.1% (1/11)
		4. Temporary decrease in productivity	9.1% (1/11)

Note: “n”: number of responses.

**Table 5:** Comparison of economic parameters between current and ideal *M. hyo* vaccines.

Data	n	Median	Mean	95%CI	Min–Max	SD	SEM
Estimated Loss Per Head, $/hd	9	3.000	2.231	1.13–3.34	0.25–4	1.436	0.479
Current Vaccines’ Price, $/hd	6	0.210	0.245	0.11–0.38	0.15–0.5	0.131	0.053
Ideal Vaccines’ Price, $/hd	9	0.750	1.028	0.52–1.54	0.25–2	0.667	0.222
Ideal price- current price, $/hd	-	0.540	0.783		-	-	-
Fold change	-	3.571	4.196		-	-	-

Note: “n”: number of responses; “-”: not available, “hd”: head.

**Table 6:** Key challenges and priorities for future *M. hyo* vaccines.

Categories	n	Specific Issues	Percentage
Limitations	6	1. Poor quality of the vaccine	66.7% (4/6)
		2. Efficacy affected by maternal antibodies	33.3% (2/6)
		3. Short duration of protection	33.3% (2/6)
Availability	6	1. Shortage of vaccine storage	33.3% (2/6)
		2. Delays in vaccine availability	16.7% (1/6)
Adverse effects	6	1. Systemic reactions	16.7% (1/6)
		2. Temporary decrease in productivity	16.7% (1/6)

Note: “n”: number of responses.

**Table 7:** Comparison of economic parameters between current and ideal *G. parasuis* vaccines.

Data	n	Median	Mean	95%CI	Min–Max	SD	SEM
Estimated Loss Per Head, $/hd	10	1.000	0.962	0.47–1.45	0.25–2.26	0.681	0.215
Current Vaccines’ Price, $/hd	5	0.500	0.570	0.17–0.97	0.15–1	0.321	0.144
Ideal Vaccines’ Price, $/hd	8	0.500	0.719	0.18–1.26	0.25–2.25	0.647	0.229
Ideal price- current price, $/hd	-	0.000	0.149		-	-	-
Fold change	-	1.000	1.261		-	-	-

Note: “n”: number of responses; “-”: not available, “hd”: head.

**Table 8:** Key challenges and priorities for future *G. parasuis* vaccines.

Categories	n	Specific Issues	Percentage
Limitations	6	1. Lack of cross-protection against new variants	33.3% (2/6)
		2. Lack of a broader spectrum of protection (e.g., Combination)	33.3% (2/6)
		3. Efficacy affected by maternal antibodies	33.3% (2/6)
Availability	6	1. Delays in vaccine availability	33.3% (2/6)
		2. High cost	33.3% (2/6)
		3. Difficult finding the correct bacterin	16.7% (1/6)
Adverse effects	6	1. Local reactions	50.0% (3/6)
		2. Temporary decrease in productivity	33.3% (2/6)
		3. Allergic reactions	16.7% (1/6)
		4. Systemic reactions	16.7% (1/6)

Note: “n”: number of responses.

**Table 9:** Summary and comparison of key factors influencing vaccine development for the top 4 prioritized bacterial diseases in the U.S.

	*S. suis*			*E. coli*			*M. hyo*			*G. parasuis*		
**Demand**												
Breadth	Major U.S. pork-producing regions + Global			Major U.S. pork-producing regions + Global			Major U.S. pork-producing regions + Global			Major U.S. pork-producing regions + Global		
Long-term utility (Residual value post-elimination)	Decades~Centuries - Eradication with current known methods is extremely difficult			Decades~Centuries - Eradication is currently considered not possible			Years~Decades - Eradication is gradually being carried out in the US			Decades~Centuries - Eradication is rarely reported		
Effectiveness of current management	Slightly~Moderately effective (2.8/5.0)			Moderately effective (3.0/5.0)			Moderately~very effective (3.4/5.0)			Slightly~Moderately effective (2.9/5.0)		
Prevalence		Morbidity	Mortality		Morbidity	Mortality		Morbidity	Mortality		Morbidity	Mortality
	Pre-wean	15.00%	6.70%	Pre-wean	23.00%	6.90%	G/F	19.00%	4.40%	Nursery	11.30%	3.70%
	Nursery	13.90%	6.50%	Nursery	25.00%	11.60%						
Est. economic impact ($ Loss/head)	~$1.25			~$1.37			~$2.23			~$0.96		
**AMR**												
Antibiotic dependence	High			High			High			High		
Phenotypic AMR prevalence	High			At a crisis point			Low~Moderate			High, varies by country		
**Vaccines**												
Effectiveness of current vaccines	Moderately effective (3.0/5.0)			Slightly~Moderately effective (2.8/5.0)			Slightly~Moderately effective (2.5/5.0)			Moderately~very effective (3.3/5.0)		
(Price of ideal vaccine)/(Price of current vaccine)	$0.83/$0.47 = 1.79 × (*)			$0.79/$0.42 = 1.88 × (*)			$1.03/$0.25 = 4.20 × (*)			$0.72/$0.57 = 1.26 × (ns)		
Primary drawbacks to overcome	- Lack of timely distribution; Takes too long to develop a farm-tailored vaccine - Lack of cross-protection			- Short duration - Inconvenient administration - Lack of cross-protection			- Poor quality - Short duration			- High cost - Lack of cross-protection - Local reaction		
Reasons for the limited effectiveness of vaccines (direct or indirect)	- Multiple serotypes, strains, and high phenotypical diversity. - Unclear antibodies needed to elicit immunity. - Lack of markers to distinguish commensal strains from virulent ones.			- Unclear mechanism for cross-reactivity of complex surface antigens (e.g., F18ab vs. F18ac). - Challenges in isolating the strains responsible for the infection. - Unclear divergences in gene transfer among strains.			- The mechanisms of interaction between hosts and bacteria remain not clearly understood, particularly regarding their pathological interaction with the respiratory tract and the immune system. - Unclear contribution of various genes to virulence. - Difficult to isolate and culture.			- Certain immunogenic processes are still unclear. - Inconsistent protection even within the same serovar. - Some strains remain non-typeable due to unclear antigenic serotyping components.		

Note: “G/F”: Grower & Finisher; “*”: a significant difference between groups is observed (*p* < 0.05); “ns”: no significance.

## Data Availability

The datasets used and/or analyzed during the current study are available from the corresponding author upon reasonable request.
